# Protein-Protein Interactions Prediction Based on Iterative Clique Extension with Gene Ontology Filtering

**DOI:** 10.1155/2014/523634

**Published:** 2014-01-22

**Authors:** Lei Yang, Xianglong Tang

**Affiliations:** School of Computer Science and Technology, Harbin Institute of Technology, Mailbox 352, 92 West Dazhi Street, Nan Gang District, Harbin 150001, China

## Abstract

Cliques (maximal complete subnets) in protein-protein interaction (PPI) network are an important resource used to analyze protein complexes and functional modules. Clique-based methods of predicting PPI complement the data defection from biological experiments. However, clique-based predicting methods only depend on the topology of network. The false-positive and false-negative interactions in a network usually interfere with prediction. Therefore, we propose a method combining clique-based method of prediction and gene ontology (GO) annotations to overcome the shortcoming and improve the accuracy of predictions. According to different GO correcting rules, we generate two predicted interaction sets which guarantee the quality and quantity of predicted protein interactions. The proposed method is applied to the PPI network from the Database of Interacting Proteins (DIP) and most of the predicted interactions are verified by another biological database, BioGRID. The predicted protein interactions are appended to the original protein network, which leads to clique extension and shows the significance of biological meaning.

## 1. Introduction

Identifying protein-protein interaction (PPI) and constructing biological networks are vital to understand the molecular function and cellular organization [1]. In recent year, protein interactions have been enriched by high-throughput biology experimental methods [2, 3]. Furthermore, there are a large number of computational methods to improve these datasets and overcome the experimental limitations of time-consuming and cost [4–6]. The categories of computational methods for predicting PPI differ among studies. For example, in paper [5], methods of predicting PPI are classified according to the structural, genomic, and biological contexts of proteins. Of them, topology methods based on PPI networks are simple to use and demand few additional information. However, the reliability based on topology methods usually fluctuates because of false positive and negative interactions in PPI network. A clique within a PPI network is an induced complete subgraph, with constituent proteins that completely interact with each other. Cliques in PPI networks are related to protein complexes and functional modules tightly and have a biological significance [6]. And components in protein complexes or functional modules are prone to interacting with each other. Thus, protein interactions can be predicted based on completing defective cliques in a PPI network. Besides, the estimation based on gene ontology (GO) annotations can enhance the accuracy of PPI predictions [7]. This is because PPIs from cliques usually have common terms in GO annotations of cellular component (CC) or molecular function (MF), due to the correlation of cliques with complexes or functional modules. Therefore, we design a predicting PPI method combining cliques and GO terms to overcome the unreliability of predicting PPI based on topology methods with the interference of noise in datasets.

Applying different GO rules, we generate two predicted PPI sets, that is, CORE and ALL which insure the quality and quantity of predictions, respectively. The proposed method is applied on DIP dataset [8]. The two predicted sets are estimated with a statistical method based on gold standard datasets [9], and the results show the effectiveness of our method. Furthermore, we introduce another dataset, BioGRID [10], recording a larger number PPIs from biological experiments to verify the correction of the predictions. As a result, most of predictions are identified with the various records of biological experiments. Appending the predictions into the PPI network, the mined cliques are close to complete complexes.

## 2. Materials and Methods

A PPI network is used to describe a set of protein pairs, and it can be modeled as a graph. We use an undirected graph *G* = (*V*, *E*) to represent a PPI network, where *V* is the set of all nodes (proteins) and *E* is the set of all edges (interactions) in graph *G*.

**Figure 1:**
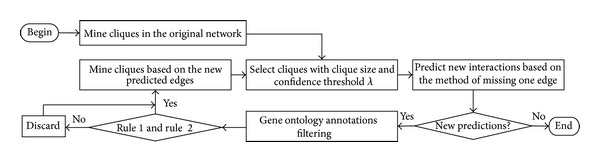
Flowchart of predicting protein interactions in a PPI network.

We propose an iterative method based on cliques and GO annotations to predict PPI (see Figure 1). First, clique mining is performed in a PPI network. Second, cliques satisfied with two conditions are selected to participate in PPI prediction. Third, the missing-one-edge method is used to predict interactions based on the mined cliques. Then, the predictions are corrected via gene ontology annotations. This process is repeated until no new prediction of PPI generates.

Mining cliques in a PPI network is a NP-complete problem according to graph computing theory [11]. However, the existing methods of mining cliques work well based on the characteristic of scale-free topology [12]. Based on an original network, the method of Gendreau et al. [13] is brought in to obtain all cliques. Then, new interactions are predicted based on these cliques.

### 2.1. Selection of Cliques Participated in PPI Prediction

Not all cliques participate in the process of predicting PPI. We set a minimum threshold of clique size. Only the cliques whose sizes are more than that threshold have the qualification to participate in PPI prediction. Furthermore, we set a clique confidence score to avoid that the selected cliques include many predicted PPIs. The clique confidence is determined by sum score of all the edges in it. That is,

(1) Cliquescore=∑e∈k-cliqueescorek(k−1)/2, where  escore={1 originalPPI0 predictedPPI.

We set a threshold *λ* of the clique score for PPI prediction. Obviously, cliques selected as the participants for predicting PPI must satisfy with the two mentioned thresholds.

### 2.2. Prediction of PPIs with Missing-One-Edge Method

We count the number of the nodes connected with the members of the known cliques. Based on a known *k*-clique, the appending of one edge extends it to (*k* + 1)-clique(s). Corresponding to the direct connection, the missing edge that links the nodes in and out of the clique to form a larger clique is a predicted interaction. As to a given *k*-clique, the description of prediction with missing-one-edge method is as below.

#### 2.2.1. Building a Candidate Set *C* to Deposit All Possible Extending Clique Nodes

*C* = *N*(*n*_1_) ∪ *N*(*n*_2_)∪⋯∪*N*(*n*_*k*_)−{*n*_1_, *n*_2_,…, *n*_*k*_}, where *n*_*i*_ is the node in *k*-clique and *N*(*n*_*i*_) represents the neighbor set of *n*_*i*_.

#### 2.2.2. Filtering Nodes in the Candidate Set *C*

*C* ← *C* − {*n* | Degree(*n*) < *k* − 1, *n* ∈ *C*}, where Degree(*n*) represents the number of the neighbors of node *n*.

#### 2.2.3. Predicting

1. Getting a node *w* in *C*.
2. Judging whether ∃*i* making *n*_*i*_ *w* ∉ *E*_all_ and ∀*j*, *n*_*j*_ *w* ∈ *E*_all_, *j* = 1,2,…, *k*, *j* ≠ *i*, where *E*_all_ = *E* ∪ {predictions}.
3. If condition (2) is satisfied, *n*_*i*_ *w* is a new predicted PPI.
4. Repeating (1) until all of the nodes in *C* have been reached.

### 2.3. Correction of Predicted PPIs by GO Terms

Proteins in a complex always have same cellular components, and proteins in a function module often perform similar biological roles. Therefore, GO terms contribute to reducing false-positive predictions [14] before the next round of clique mining based on the predictions. We set two GO rules as follows.

**Rule 1**

Two proteins in the predicted interaction should have a common cellular component at least.

**Rule 2**

Two proteins in the predicted interaction should have a common molecular function at least.

If only Rule 1 is used to filter the predicted PPIs, a loose PPI prediction set (ALL) is gotten. If both Rules 1 and 2 are applied to filter the predicted PPIs, a tight prediction set (CORE) is obtained.

### 2.4. Clique Generation Based on New Predicted Interactions

**Figure 2:**
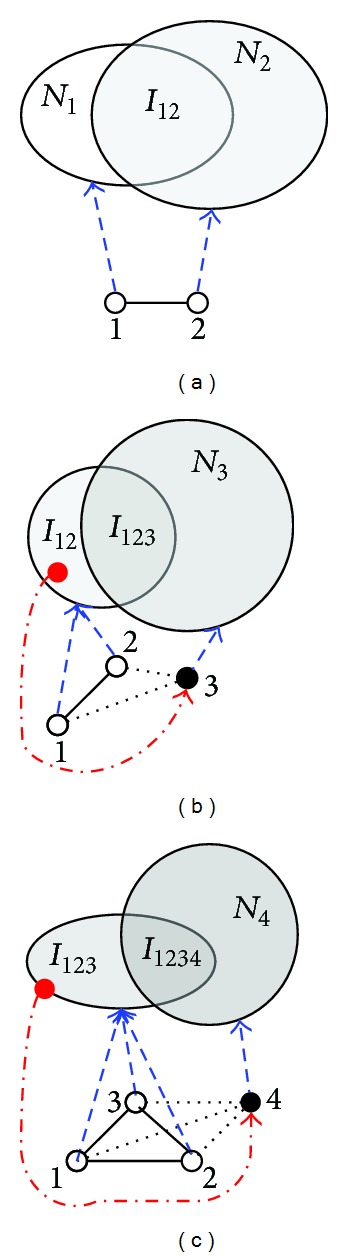
An illustration of mining cliques based on predicted protein pairs. (a) Components of intersection *I*_12_ between the neighbors of node 1 and node 2 are candidates to form 3 cliques. (b) Node 3 derived from *I*_12_ with nodes 1 and 2 forms 3 cliques. The neighbors of node 3 intersect *I*_12_ and construct the candidate set *I*_123_ of 4 cliques. (c) The node in the *I*_123_ composes of nodes 1, 2, and 3 to form 4 cliques. The neighbors of node 4 derived from *I*_123_ intersect *I*_123_ and construct the candidate set *I*_1234_ for forming 5 cliques. This procedure is repeated until the maximum clique is found based on node 1 and node 2.

After we obtain the predicted PPIs of the first round, the new predictions are merely related with the cliques including interactions just predicted in the previous round. Therefore, we design an appropriate method to generate cliques only based on the predicted interactions to minimize the computational cost. Mining cliques is a recursively bottom-up procedure, as illustrated in Figure 2. First, all 3 cliques are found out based on the new predicted PPIs (see Figure 2(a)). Then, all 4 cliuqes are found out based on the 3 cliques (see Figures 2(b) and 2(c)). In turn, *k*-cliques are mined based on the (*k* − 1)-cliques until the maximum cliques are found.

## 3. Results and Discussion

### 3.1. Performance on DIP Dataset

Our method is applied on a dataset of PPIs in *Saccharomyces cerevisiae* downloaded from the Database of Interacting Proteins (DIP, version of 2010/6/14) [8]. DIP is generally acknowledged as an excellent data source containing experimentally determined protein-protein interaction. This version dataset contains 26718 interactions. Getting rid of proteins of self-interacting, we achieve 4997 protein nodes and 23233 protein-protein interaction pairs from DIP database.

The maximum size of mined clique is 10. According to this maximum size, we set the minimum size of clique participating in predicting PPI to be 6. The threshold of clique confidence score is set to be 0.7. Based on the PPI network of the original DIP dataset, 442 PPI predictions are generated. Then, according to the cliques derived from these predictions and different GO rules, CORE and ALL are generated, respectively. CORE includes 352 predictions and ALL contains 874. Adding these predictions into the original dataset, the size of the maximum clique is enlarged to 16 and the number of small cliques is reduced, that is, consolidation of smaller cliques into larger ones.

### 3.2. Estimation with BioGRID Dataset

We expect predictions in CORE and ALL to be validated by the other records of biological experiments. Database BioGRID collects sufficient and reliable data in *Saccharomyces cerevisiae* from primary literatures. Therefore, we compare CORE and ALL with the dataset of BioGRID (version 3.2.98) and find that the overlaps between them are very high. Of 352 predictions in CORE, 323 are found in BioGRID and the overlapping ratio is near 92%. Of 874 predictions in ALL, 774 are found in BioGRID and the overlapping ratio is nearly 89%. The process of predicting and validating PPI in each round is shown in Tables 1 and 2.

### 3.3. Estimation with Statistics

A statistical method based on gold standard (GS) datasets is introduced to estimate the PPI predictions [9], where the GS is essentially a likelihood ratio *L*, that is, *L* = (*P*_+_/*G*_+_)/(*P*_−_/*G*_−_). *P*_+_ is the number of repaired edges of cliques contained in the true-positive GS set. *P*_−_ is the number in the true-negative GS set. *G*_+_ is the number of the true-positive set of GS and *G*_−_ is the number of the true-negative set of GS. Jansen et al. [9] have pointed out that the PPI predictions are acceptable with *L* > 600. *G*_+_ and *G*_−_ are constant and equal to 8250 and 2705844, respectively. The estimation of value *L* in CORE and ALL is listed in Table 3. The *L* values of two predicted PPI sets are more than the acceptable threshold. The predicting quality of CORE is obviously better than ALL.

### 3.4. Comparison with Other Methods

Based on a network topology to predict protein interactions, clique methods are usually more reliable and accurate than clustering methods in the dense region of protein interaction network. Therefore, we select two methods based on cliques to compare with our method. The first is DC method of Yu et al. [15], which predicts interactions in protein interaction network by completing defective cliques. It is similar with our method in the first step to generate predicted PPI candidate set. DC method predicts PPIs only once; yet, our method generates them step by step with GO filtering. The second is the molecular complex detection algorithm (IPC-MCE) [16], which uses node extension approach based on the interaction probability with the known cliques to extend cliques in dense regions. IPC-MCE algorithm can also be viewed as a method of predicting interactions, if we consider all proteins in a predicted complex to interact with each other.

**Figure 3:**
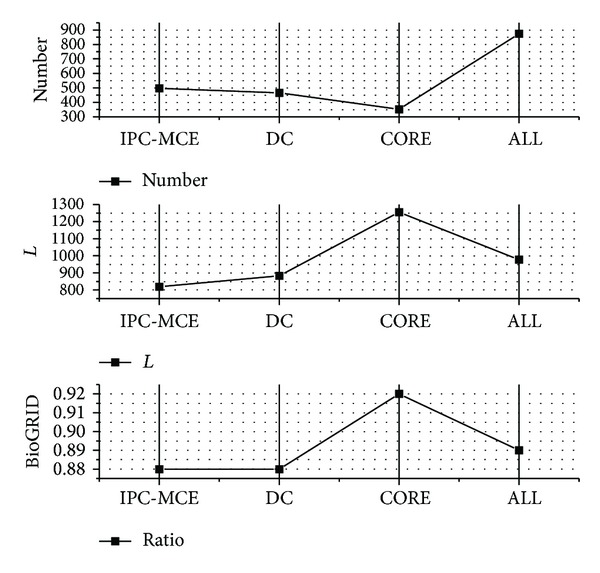
ALL has the largest number of predicted pairs. The estimation results with value *L* and BioGRID are better than the other compared methods. CORE has the largest accurate but small number. These predictions in CORE pursue high quality by sacrificing the predicted quantity.

Using DC method, the parameters of nonoverlapping size and overlapping size of a clique are, respectively, sets 3 and 4 [15]. The lower size of non-overlapping parts and the higher size of overlaps can achieve good performance of predictions. Hence, in this paper, we set them 3 and 6, respectively. For IPC-MCE algorithm, we set the probability threshold *t* to 0.8. These parameters of two compared methods are set to be stricter than applied in the original papers for getting better results. DC method predicts 465 protein-protein interactions and 407 are hit in the BioGRID dataset. Furthermore, value *L* is estimated in GS datasets. There are 70 in true positives and 26 in true negatives based on gold standard datasets, and the value *L* is 883. IPC-MCE algorithm generates 496 predicted interactions and 436 are hit in BioGRID. And 79 are in true-positive dataset and 31 are in true-negatives based on gold standard datasets. The value *L* is 836. The results compared between various methods are shown in Figure 3.

### 3.5. Intersection of CORE and ALL

**Figure 4:**
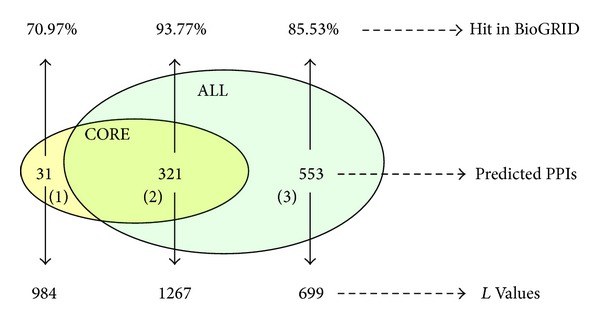
The overlap and difference between CORE and ALL are estimated with the dataset BioGRID and statistical likelihood benchmark *L*.

There are 321 interactions between CORE and ALL, as shown in section (2) of Figure 4. The remaining areas of CORE and ALL are represented with sections (1) and (3), respectively. The predictions in section (2) are validated to be better than PPIs in CORE and ALL. Therefore, the overlapping interactions between two sets have high reliability and are more suitable to complement the PPI datasets from biological experiments.

### 3.6. Assessment of Cliques Extended by Predicted PPIs

**Figure 5:**
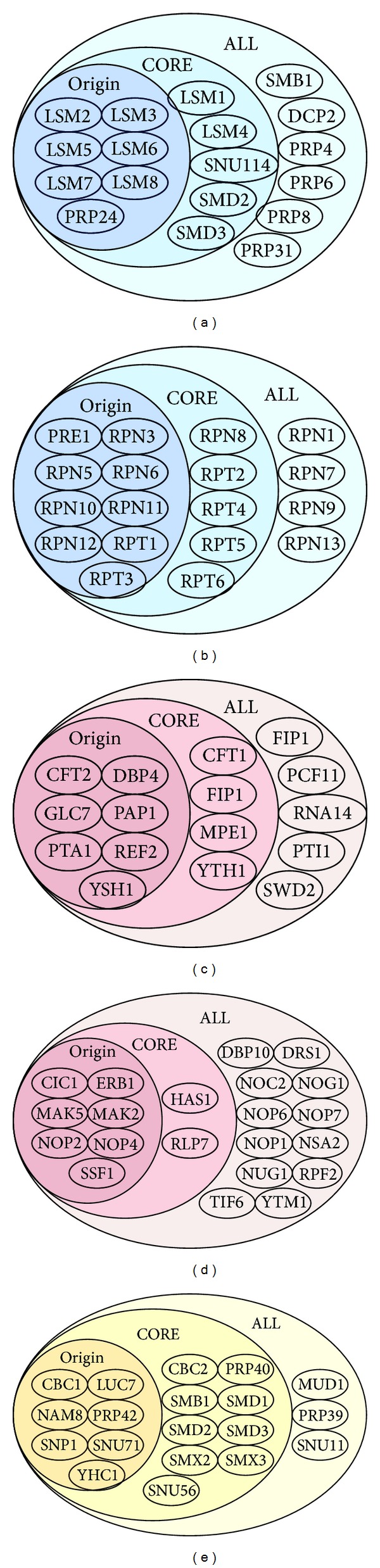
Cliques extended by the predicted PPIs from CORE and ALL have high functional significance and physical meaning.

The predicted PPIs using our methods can well complement missing interactions to extend potential cliques (see Figure 5). We construct a component set of a clique mined from original DIP database within the Origin area. The Core area is extended by the predicted PPIs from CORE based on the Origin area. The All area is extended by the predicted PPIs from ALL based on the Origin area. We find that many extended cliques exhibit better quality than original cliques under GO annotations. In Figure 5(a), a 7-clique mined in the original network is extended to a 12-clique, then extended to an 18-clique. The number of U4/U6 × U5 tri-snRNP complex included in clique is increased from 6 to 11, then to 16. The components of original cliques are improved in the protein complexes. In Figure 5(b), a 9-clique mined in the original network is extended to a 14-clique, then extended to an 18-clique. The number of complex components included in the clique is increased from 9 to 14, then to 18. In Figure 5(c), a 7-clique mined in the original network is extended to an 11-clique, then extended to a 16-clique. The number participating in the mRNA processing included in the clique is increased from 6 to 10, then to 15. The components of original cliques increase in the same biological process. In Figure 5(d), a 7-clique mined in the original network is extended to a 9-clique, then extended to a 21-clique. The number participating in the ribosome biogenesis process included in the clique is increased from 7 to 9, then to 19. In Figure 5(e), a 7-clique mined in the original network is extended to a 16-clique, then extended to a 19-clique. The number participating in RNA binding included in clique is increased from 6 to 15, then to 16. The components of the original clique are extended in the molecular function.

## 4. Conclusions

Cliques in the PPI network are good resource and worth to study properly. Predicting PPIs by recursively extending cliques results in a quantity increase of predicted PPIs. And the quality of predictions is significantly improved via combining GO annotations with clique-based method. Obviously, iteratively finding cliques is a time-consuming task. Especially, the PPI network is frequently extended by new PPIs. Therefore, we design a special and novel method based only on the new predictions to mine cliques. This method is feasible and efficient in practice, which decreases the solution searching space and obtains high performance. Our work also partly solves the problem of missing cliques due to the false negative interactions. The clique size increases with continuously appending the predicted PPIs into the original network. Most enlarged cliques show good biological meaning with GO annotations. Besides, the predicted PPIs can complement data defection associated with protein complexes which are too large to detect all of its components with biological experiments. In the future, our methods on predicting PPIs and extending cliques will be applied to other PPI networks, such as human protein network, to help us associate disease relationship in the cliques.

## Figures and Tables

**Table 1:** Prediction steps of CORE validated with BioGRID.

Round	Max. clique	Predicted PPIs	PPIs after GO	In BioGRID	Confirmed ratio
1	10	442	149	143	0.96
2	13	421	130	114	0.88
3	15	154	64	57	0.89
4	15	29	8	8	1
5	11	6	1	1	1

**Table 2:** Prediction steps of ALL validated with BioGRID.

Round	Max. clique	Predicted PPIs	PPIs after GO	In BioGRID	Confirmed ratio
1	10	442	368	335	0.91
2	16	539	357	305	0.85
3	15	161	122	109	0.89
4	15	21	19	17	0.89
5	13	4	4	4	1
6	9	4	4	4	1

**Table 3:** *L* value estimated by the GS sets.

	Predictions	*P*_+_	*P*_−_	*L*
CORE	352	88	23	1255
ALL	874	134	45	977
